# The Thermal Dose of Photothermal Therapy Generates Differential Immunogenicity in Human Neuroblastoma Cells

**DOI:** 10.3390/cancers14061447

**Published:** 2022-03-11

**Authors:** Palak Sekhri, Debbie K. Ledezma, Anshi Shukla, Elizabeth E. Sweeney, Rohan Fernandes

**Affiliations:** 1 George Washington Cancer Center, Department of Medicine, School of Medicine and Health Sciences, George Washington University, Washington, DC 20052, USA; palaksekhri@gwu.edu (P.S.); dkledezma@gwmail.gwu.edu (D.K.L.); anshi.shukla1610@gmail.com (A.S.); 2 The Institute for Biomedical Sciences, School of Medicine and Health Sciences, George Washington University, Washington, DC 20052, USA; 3 George Washington Cancer Center, Department of Biochemistry & Molecular Medicine, School of Medicine and Health Sciences, George Washington University, Washington, DC 20052, USA

**Keywords:** photothermal therapy, thermal dose, immunogenicity, immunogenic cell death, Prussian blue nanoparticles, neuroblastoma, nanoimmunotherapy, MYCN amplification

## Abstract

**Simple Summary:**

Photothermal therapy (PTT) is an effective thermal therapy for treating tumors. PTT has been combined with immunotherapy in various preclinical cancer models showing promising treatment outcomes. However, in these studies, PTT has primarily been utilized for maximizing tumor cell death. Previously, we observed that based on the “thermal dose” applied, PTT can generate dramatically different responses from the immune system when tested in cellular and animal models. Here, we sought to provide a framework to systematically assess the effect of PTT-based thermal doses on the immunogenic correlates of treated tumors as a measure of the effectiveness of PTT in eliciting an antitumor immune response. In human neuroblastoma tumor cells in vitro, we determined specific phenotypic markers, which demonstrated that SH-SY5Y cells were more responsive to PTT-based thermal dose compared with LAN-1 cells, which possess a high-risk phenotype. Our findings suggest the importance of conducting tumor thermal dose-responsiveness studies in vitro as an early measure of PTT effectiveness against a specific tumor.

**Abstract:**

Photothermal therapy (PTT) is an effective method for tumor eradication and has been successfully combined with immunotherapy. However, besides its cytotoxic effects, little is known about the effect of the PTT thermal dose on the immunogenicity of treated tumor cells. Therefore, we administered a range of thermal doses using Prussian blue nanoparticle-based photothermal therapy (PBNP-PTT) and assessed their effects on tumor cell death and concomitant immunogenicity correlates in two human neuroblastoma cell lines: SH-SY5Y (*MYCN*-non-amplified) and LAN-1 (*MYCN*-amplified). PBNP-PTT generated thermal dose-dependent tumor cell killing and immunogenic cell death (ICD) in both tumor lines in vitro. However, the effect of the thermal dose on ICD and the expression of costimulatory molecules, immune checkpoint molecules, major histocompatibility complexes, an NK cell-activating ligand, and a neuroblastoma-associated antigen were significantly more pronounced in SH-SY5Y cells compared with LAN-1 cells, consistent with the high-risk phenotype of LAN-1 cells. In functional co-culture studies in vitro, T cells exhibited significantly higher cytotoxicity toward SH-SY5Y cells relative to LAN-1 cells at equivalent thermal doses. This preliminary report suggests the importance of moving past the traditional focus of using PTT solely for tumor eradication to one that considers the immunogenic effects of PTT thermal dose to facilitate its success in cancer immunotherapy.

## 1. Introduction

Photothermal therapy (PTT) is a preclinical method of treating tumor cells in vitro and in vivo via light-absorbing nanoparticles activated by a matching wavelength light source, typically a near infrared laser [1,2]. The nanoparticles absorb incident light, converting it to heat energy through a process called photothermal conversion [3,4]. This resultant heating generates hyperthermia or ablation of treated cancer cells in vitro or the tumor microenvironment in vivo, and has been shown to be effective in killing cancer cells both in cell culture and in animal models, respectively [5,6,7].

Several groups including our own (using Prussian blue nanoparticles; PBNPs) have synthesized nanoparticle-based photothermal agents with high photothermal conversion efficiencies that can generate tumor cell killing in several types of cancer [5,6,8,9,10,11,12,13,14]. In the past decade, the focus has shifted from using PTT as a single therapy to using PTT in combination with immunotherapy such as immune checkpoint inhibitors, immune adjuvants (e.g., toll-like receptor agonists), or adoptively transferred immune effector cells [6,15,16,17,18]. Implicit in these “nanoimmunotherapy” combinations is the understanding that PTT generates an antitumor immune effect in addition to tumor cell killing. However, in most of these studies, including our own, PTT was applied under conditions (i.e., using nanoparticle concentrations and laser powers) to maximize tumor cell death without necessarily considering if those maximal tumor cell death conditions were optimal for potentiating antitumor immunity. In 2018, we published a study wherein we observed that PTT using PBNPs (PBNP-PTT) administered within an optimal thermal dose window, which was lower than the highest thermal dose tested, generated immunogenic cell death (ICD) and increased survival in a murine Neuro2a neuroblastoma vaccination model [19]. ICD is a cell death mechanism characterized by the engagement and stimulation of dendritic cells (DCs) and T cells against cancer cells [19,20,21,22,23]. These findings suggested the importance of studying the effect of the applied thermal dose of PTT on the immunogenicity correlates of treated tumors to maximize the benefit of combining PTT with immunotherapy.

Building upon our earlier observations and similar observations by other groups [24,25], in this study we investigated the effects of thermal dose generated by PBNP-PTT on the immunogenicity correlates of human neuroblastoma cells in vitro. Neuroblastoma remains a deadly pediatric cancer despite incremental improvements to the standards of care [26]. Patients with high-risk neuroblastoma have a five-year survival prognosis of only 40–50%, thus necessitating the development of novel and effective treatment strategies for this patient population [27,28]. We modeled pediatric neuroblastoma using two human cell lines, SH-SY5Y and LAN-1. These cell lines differ in their *MYCN* oncogene expression. *MYCN* encodes for the N-myc proto-oncogene, a protein functionally involved in tumorigenesis [29,30,31]. *MYCN* amplification is associated with high-risk neuroblastoma, and is characterized as a genetic marker for neuroblastoma risk stratification [32,33,34,35]. SH-SY5Y is a *MYCN*-non-amplified cell line, while LAN-1 is *MYCN*-amplified [36,37]. Therefore, LAN-1 cells represent a higher-risk neuroblastoma subtype than SH-SY5Y cells. In addition to *MYCN* amplification, LAN-1 cells bear a *p53* mutation [38], increasing their high-risk phenotype.

**Figure 1:**
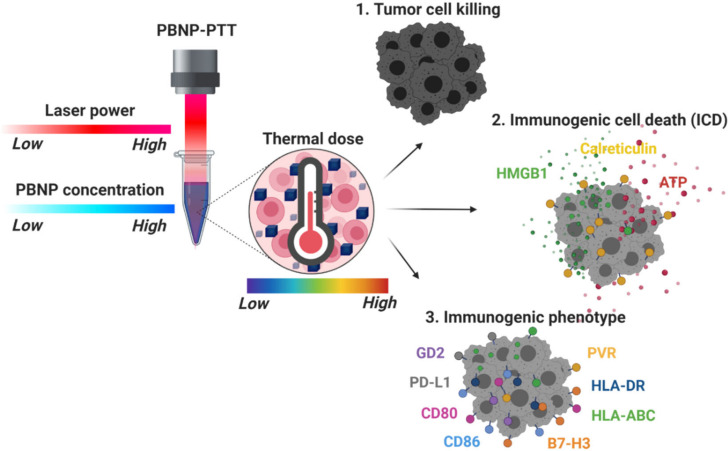
Schematic of the study. The thermal dose generated by PBNP-PTT was controlled by varying the PBNP concentration and near infrared laser power, independently. Subsequently, tumor cell death, immunogenic cell death (ICD), and cell surface markers associated with immunogenicity were measured on neuroblastoma cells as a function of PBNP-PTT-administered thermal dose.

We selected the PBNP-PTT conditions to generate various thermal dose ranges (1.8–11.3 log(∑CEM43); spanning final temperatures of ~32–80 °C) administered to the SH-SY5Y and LAN-1 neuroblastoma cell lines, and identified metrics to assess the cytotoxicity and immunogenicity correlates as a function of PBNP-PTT thermal dose (Figure 1). Specific PBNP-PTT thermal doses were achieved by varying PBNP concentration and near-infrared laser power. To calculate the thermal dose administered during PBNP-PTT, we used the method and equation first described by Sapareto and Dewey [39]. To assess the cytotoxicity and immunogenicity correlates, we measured the viability of the cells, the induction of ICD, and the phenotypic changes (i.e., expression of costimulatory molecules, immune checkpoint molecules, major histocompatibility complexes, an NK cell-activating ligand, and a neuroblastoma-associated antigen) resulting from each thermal dose. Next, we compared the immunogenic effects of the PBNP-PTT-administered thermal doses on *MYCN-*non-amplified SH-SY5Y cells versus *MYCN-*amplified LAN-1 cells. Finally, we conducted functional studies where T cells were co-cultured with either SH-SY5Y or LAN-1 cells treated with various PBNP-PTT thermal doses (low to high). We assessed the cytotoxicity of the T cells toward the tumor cells as a function of the thermal doses. 

Through these studies, we sought to provide a framework for assessing the effect of the thermal dose of PTT not only on tumor cell killing but also on the immunogenicity correlates of the treated tumor cells. Conducting these important in vitro studies will facilitate the design of in vivo studies for a specific tumor where PTT is administered at the optimal thermal dose to maximize the antitumor immune benefit of this thermal therapy against that tumor.

## 2. Materials and Methods

### 2.1. Cells

The human neuroblastoma cell line SH-SY5Y was obtained from American Type Culture Collection (ATCC, Manassas, VA, USA). The human neuroblastoma cell line LAN-1 was purchased from Sigma-Aldrich (St. Louis, MO, USA). SH-SY5Y cells were cultured in 44% DMEM (Gibco, Carlsbad, CA, USA), 44% F-12K (Gibco), 10% heat-inactivated FBS (Cytiva, Marlborough, MA, USA), 1% glutamax (Gibco), and 1% penicillin-streptomycin (Gibco). LAN-1 cells were cultured in RPMI-1640 (Gibco) supplemented with 10% heat-inactivated FBS (Cytiva), 1% glutamax (Gibco) and 1% penicillin-streptomycin (Gibco). SH-SY5Y cells are *MYCN*-non-amplified, thereby potentially representing a lower risk neuroblastoma classification; LAN-1 cells are *MYCN*-amplified, thereby representing a high-risk neuroblastoma subtype [36,37]. 

### 2.2. PBNP Synthesis

Potassium hexacyanoferrate (II) trihydrate (K_4_[Fe(CN)_6_]·3H_2_O) and iron (III) chloride hexahydrate (Fe(Cl)_3_·6H_2_O) were purchased from Sigma-Aldrich. All synthetic procedures were conducted using deionized (DI) water for synthesis purchased from Sigma-Aldrich (catalog #848333). Prussian blue nanoparticles (PBNPs) were synthesized using a scheme as described previously [6,40,41], and characterized for uniformity (Figure S1). Briefly, an aqueous solution of 1.0 mM FeCl_3_·6H_2_O in 20 mL of DI water was added under vigorous stirring to an aqueous solution containing 1.0 mM of K_4_Fe(CN)_6_·3H_2_O in 20 mL of DI water. After stirring for 15 min, the precipitate was isolated by centrifugation in equal parts DI water and acetone (10,000× *g* for 10 min) at room temperature, and rinsed by sonication (5 s, 40% amplitude) in DI water using a Q500 sonicator (QSonica LLC, Newton, CT, USA). The isolation and rinsing steps were repeated three times before the particles were resuspended by sonication in DI water. To measure the physical properties of the nanoparticles, size (hydrodynamic diameter) and charge distributions (zeta potential) were measured using dynamic light scattering on a Zetasizer Nano ZS (Malvern Instruments, Malvern, UK).

### 2.3. Photothermal Therapy

Three million SH-SY5Y or LAN-1 cells were suspended in 500 μL media with 0, 0.06, 0.1, or 0.15 mg/mL PBNPs. The samples were then illuminated by the near-infrared laser (808 nm; Laserglow Technologies, Toronto, Canada) for 10 min at varied powers (0.75, 1.0, or 1.5 W) to administer a range of thermal doses. Power was confirmed using a power meter (Thorlabs, Newton, NJ, USA). Temperatures of the cell suspensions were measured using a thermal camera (FLIR, Arlington, VA, USA), and recorded every minute for 10 min. The thermal doses were calculated using the formula established previously in the literature: ∑CEM43 °C = ∆*t* × *R*^(43−*T*)^ where ∆*t* is the time interval, T is the average temperature during time interval, and R is the constant 0.25 for *T* < 43 °C and 0.5 for *T* > 43 °C [39]. The treated cells were then plated in a 6-well plate and incubated at 37 °C for 24 h before further analysis was performed. 

### 2.4. Cell Phenotypic Analysis

#### 2.4.1. Cell Surface Marker Analysis

Antibodies were purchased from Biolegend (San Diego, CA) and Abcam (Cambridge, UK). After 24 h incubation at 37 °C post-PBNP-PTT, cells were harvested and stained with Zombie Aqua Fixable viability dye (Biolegend, #423102), blocked with human TruStain Fc block (Biolegend, #422302), and stained with fluorescent antibodies against calreticulin (Abcam, #ab83220), CD80 (Biolegend, #305238), CD86 (Biolegend, #305428), PD-L1 (Biolegend, #329714), B7-H3 (Biolegend, #351010), HLA-ABC (Biolegend, #311432), HLA-DR (Biolegend, #307633), PVR (Biolegend, #337628), and GD2 (Biolegend, #357308). Flow cytometry was performed using the Cytek Aurora cytometer (Cytek Biosciences, Fremont, CA, USA), and cytometric analysis was done using FlowJo software (Ashland, OR, USA) to assess the immunogenicity correlates described above as a function of PBNP-PTT thermal dose. 

#### 2.4.2. ATP Release Analysis

For estimation of intracellular ATP (a correlate of ICD [23]), cells were harvested 24 h after in vitro PBNP-PTT, washed with 1x PBS, and mixed with the ATP reagent from the CellTiter-Glo Luminescent Cell Viability Assay (Promega, Madison, WI, USA). Luminescence was then measured using a SpectraMax i3X microplate reader (Molecular Devices, San Jose, CA, USA), whereupon luminescence was correlated to intracellular ATP as a measure of ATP released from the cells, a critical component of ICD induction.

#### 2.4.3. HMGB1 Release Analysis

24 h post treatment, cell supernatants were collected from each treatment condition and centrifuged at 10,000 rpm for 3 min at 4 °C. The supernatants were collected and stored at −80 °C. HMGB1 release from cells was measured using an ELISA kit purchased from Chondrex Inc. (Woodinville, WA, USA) and the manufacturer’s recommendation was followed. Briefly, the 96-well ELISA plate was coated with the capture antibody overnight at 4 °C. The supernatant samples were thawed, both samples and standards were diluted 1:1 in a sample dilution buffer, and were added to the washed plate in duplicate. Next, the detection antibody was added and the plate was incubated at 37 °C for 1 h. Subsequently, the ELISA plate was transferred to a 4 °C refrigerator overnight. Next, diluted streptavidin peroxidase solution was added to the wells and incubated for 30 min at room temperature. The wells were washed and TMB solution was added to the plate. After incubation for 30 min at room temperature, a stop solution was added and the absorbance was measured at 450 nm using a Spectramax i3X microplate reader (Molecular Devices). The media alone condition was used as a blank for the laser condition (not containing PBNPs); the PBNP alone condition was used as a blank for all PBNP-PTT conditions. Blanks were subtracted from each sample to accurately estimate the concentration of HMGB1 released from treated cells, a key ICD biochemical correlate [23].

### 2.5. Immunogenicity Correlates as a Function of PTT Thermal Dose Analysis

Cell phenotypes were plotted as a function of thermal dose to interpret the magnitude of immunogenic responses in SH-SY5Y and LAN-1 cells resulting from varied PBNP-PTT thermal doses. For the measurement of ATP, the intracellular ATP content of untreated cells was considered to be 100%, and treatment conditions were normalized to that value. For the measurement of extracellular HMGB1, the laser alone condition was normalized to media treatment, and the PBNP-PTT conditions were normalized to treatment with PBNPs (in the absence of laser) to account for a potential interference in the absorbance reading by the presence of PBNPs. For the expression of immunogenicity correlates measured by flow cytometry, the median fluorescence intensity (MFI) resulting from each thermal dose was normalized to the condition where cells were treated with the laser alone (thermal dose <0 log(∑CEM43)). By normalizing the data to internal controls, we illustrate the differences in responsiveness of the two cell lines by their differed slopes on the thermal dose versus immunogenicity correlate graphs. 

### 2.6. Studies Involving Peripheral Blood Cells

#### 2.6.1. Peripheral Blood Sourcing and Culture

Healthy donor peripheral blood leukopaks were purchased from AllCells (Alameda, CA, USA) along with the HLA report. Peripheral blood mononuclear cells (PBMCs) were isolated by density gradient centrifugation using lymphocyte-separation media (LSM; Corning, Corning, NY). PBMCs were cryopreserved for future use. Dendritic cells were grown in dendritic cell media (CellGenix, Freiburg im Breisgau, Germany) supplemented with 1% Glutamax (Gibco) (DC media). T cells were cultured in CTL media: 46.5% Click’s media (Sigma-Aldrich, Burlington, MA, USA), 46.5% RPMI-1640 (Gibco), 5% human AB serum (Gemini Bio-products), 1% glutamax (Gibco), and 1% penicillin streptomycin (Gibco).

#### 2.6.2. Generation of T Cells 

T cell lines were generated from PBMCs that were HLA-matched to SH-SY5Y and LAN-1 cells using monocyte-derived dendritic cells (DCs) based on a previously established protocol [42]. Specifically, donors were chosen based on HLA-A matching (HLA-A*24 for SH-SY5Y cells, and HLA-A*02 for LAN-1 cells). Briefly, monocytes were isolated by CD14 isolation MACS beads kit (Miltenyi Biotec, Bergisch Gladbach, North Rhine-Westphalia, Germany) per the manufacturer’s instructions. The isolated cells were cultured in DC media in the presence of IL-4 (1000 U/mL) and granulocyte-macrophage colony-stimulating factor GM-CSF (800 U/mL) (R&D Systems, Minneapolis, MN) cytokines. One day prior to stimulation, DCs for each donor were pulsed with tumor cell lysate generated using multiple freeze-thaw cycles using a dry ice-ethanol mixture and a water bath maintained at 37 °C [43]. Cell death was confirmed using a trypan blue assay. DCs were then matured with GM-CSF, TNF-a, IL-1b, IL4, IL-6, GM-CSF, INFγ, IL-4 (R&D Systems), and lipopolysaccharide (LPS; Sigma-Aldrich) overnight. Following maturation, CD14 negative PBMCs were thawed and stimulated with harvested DCs at a 1:5 (DC: T cell) ratio in CTL media supplemented with IL-6, IL-7, IL-12, and IL-15 (R&D Systems). Cells were incubated at 37 °C in an incubator and were expanded and fed with fresh cytokines as necessary. Subsequent stimulation was performed in a similar way. On day 23, cells were harvested for the functional co-culture assays.

#### 2.6.3. Assessing T Cell Cytotoxicity toward PBNP-PTT-Treated Tumor Cells 

The target cells (SH-SY5Y and LAN-1) were stained with CellTrace^TM^ Far Red using a cell proliferation kit (ThermoFisher Scientific, Waltham, MA, USA). Labeled tumor cells were subjected to PBNP-PTT at various thermal doses and immediately co-cultured with their corresponding T cells at a 1:1 effector:target cell ratio. Cells were cultured for 4 h, collected by centrifugation, and resuspended in equal volumes of buffer across all samples. Cells were stained with Zombie Green Fixable viability dye (Biolegend, #423112), blocked with human TruStain Fc block, and stained with CD3 (Biolegend, #300326) to distinguish T cells. Flow cytometry was conducted in duplicate in a 96-well plate, whereupon equal volume was collected from each sample and analyzed in the flow cytometer. T cell-mediated cytotoxicity was calculated by determining the number of live target cells (gating CellTrace Far Red positive and Zombie green negative populations). These values were normalized to the number of live target cells after respective treatments in the absence of T cells. The samples were run on a Cytoflex flow cytometer (Beckman Coulter, Brea, CA, USA). 

### 2.7. Statistical Analysis

Statistical analyses were performed using Graphpad PRISM software (San Diego, CA, USA). Statistical significance was determined by ordinary one-way ANOVA using Dunnett’s multiple comparison test. For the T cell co-culture analysis, a two-way ANOVA using Šidák multiple comparisons test was used. Values were considered statistically significantly different when *p* values were less than 0.03. * indicates a *p* value of <0.03, ** indicates a *p* value of <0.002, *** indicates a *p* value of <0.0002, and **** indicates a *p* value of <0.0001. 

## 3. Results

### 3.1. Laser Power and PBNP Concentration Controls PBNP-PTT Thermal Dose

**Figure 2:**
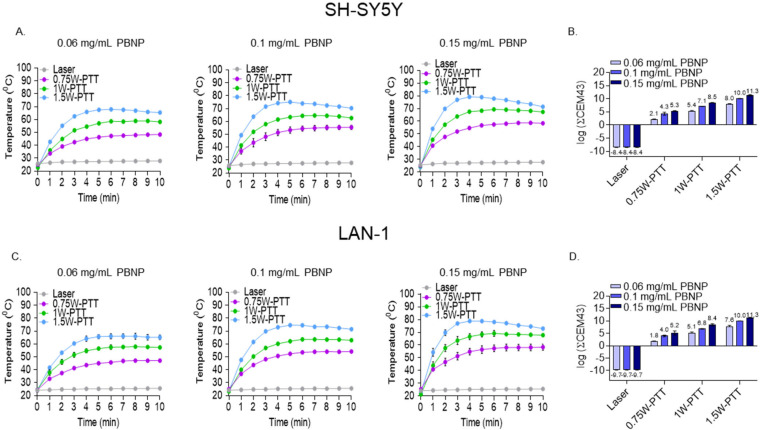
PBNP-PTT-administered thermal dose is controlled by PBNP concentration and laser power. Three million (**A**,**B**) SH-SY5Y or (**C**,**D**) LAN-1 human neuroblastoma cells were exposed to 0.75, 1, and 1.5 W laser power in combination with 0 (Laser), 0.06, 0.1, or 0.15 mg/mL PBNPs. (**A**,**C**) Time-temperature graphs represents the cell temperatures recorded for each condition at 1 min intervals for a total of 10 min. (**B**,**D**) Thermal doses applied to cells were calculated from the time-temperature curves for each condition.

To establish a range of thermal doses to test in vitro, PBNP-PTT dosing was controlled by independently varying both the PBNP concentration added to the cells and the applied near infrared laser power. First, PBNPs were synthesized and quality control attributes were analyzed. PBNPs were measured to have an average hydrodynamic diameter of 55 nm and a surface charge of −39 mV (Figure S1). PBNP-PTT was administered to SH-SY5Y or LAN-1 cells under varied PBNP concentrations and laser power conditions as described. SH-SY5Y cells treated with each PBNP concentration heated in a laser power-dependent manner; as laser power increased, cell temperatures increased over the ten minutes of heating (Figure 2A). These time-temperature curves were converted to cumulative equivalent minutes at 43 °C (∑CEM43), a parameter used to quantify the thermal dose [39], which confirmed that the thermal dose generated by PBNP-PTT was both PBNP concentration- and laser power-dependent (Figure 2B). Thermal dose also directly correlated with cytotoxicity of SH-SY5Y cells; as the thermal dose increased, SH-SY5Y cell viability decreased in a thermal dose-dependent manner, with a maximal killing of 96% at a thermal dose of 11.3 log(∑CEM43) (Figure S2). These trends were consistent in LAN-1 cells; both PBNP concentration and laser power increased the temperature of LAN-1 cells over ten minutes of heating (Figure 2D), and generated PBNP concentration- and laser power-dependent increases in thermal dose (Figure 2E). Similar to SH-SY5Y cells, increased heating caused lower LAN-1 cell viability in a thermal dose-dependent manner, with a maximal killing of 98% at a thermal dose of 11.3 log(∑CEM43) (Figure S2).

### 3.2. PBNP-PTT Generates ICD in Human Neuroblastoma Cells In Vitro

**Figure 3:**
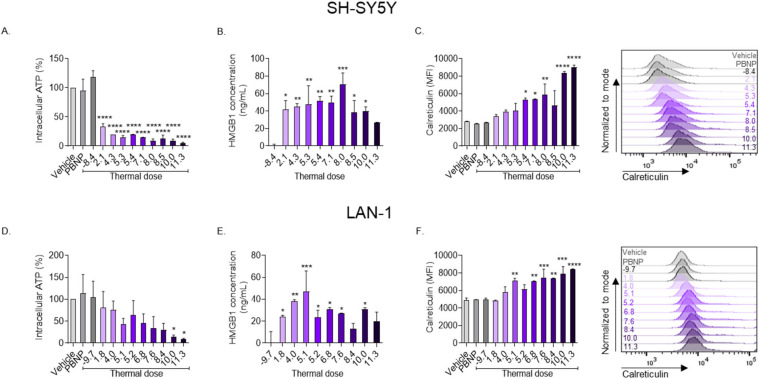
PBNP-PTT generates a thermal dose window of immunogenic cell death in SH-SY5Y and LAN-1 cells in vitro. Three million (**A**–**C**) SH-SY5Y or (**D**–**F**) LAN-1 cells were exposed to various thermal doses using PBNP-PTT. After 24 h, cells were analyzed for (**A**,**D**) intracellular ATP, (**B**,**E**) HMGB1 release, and (**C**,**F**) surface calreticulin expression, represented as median fluorescence intensity (MFI). Inset values in the histograms denote the thermal dose. The extent of ICD as measured by its correlates is more pronounced in SH-SY5Y cells compared with LAN-1 cells. Ordinary one-way ANOVA was used to calculate significance between vehicle and different thermal doses and laser alone for HMB1 analysis. *n* = 2/group; * *p* < 0.03, ** *p* < 0.002, *** *p* < 0.0002, **** *p* < 0.0001.

To determine whether the thermal doses generated during PBNP-PTT elicited ICD in the neuroblastoma cells in vitro, the consensus biochemical correlates for ICD (i.e., release of intracellular ATP and HMGB1, and upregulation of cell surface calreticulin [23]) were measured after treatment. PBNP-PTT generated a thermal dose-dependent decrease in intracellular ATP of SH-SY5Y cells, suggesting its release (Figure 3a), with <10% intracellular ATP remaining after treatment with thermal doses ≥8.0 log(∑CEM43). Concurrently, PBNP-PTT generated an increase of released HMGB1 at thermal doses ≥2.1 log(∑CEM43), with the maximal release of HMGB1 occurring at a thermal dose of 8.0 log(∑CEM43) (Figure 3B). PBNP-PTT also caused an increase in SH-SY5Y cell surface calreticulin expression in a thermal dose-dependent manner, with the maximal expression of calreticulin occurring upon treatment with a thermal dose of 11.3 log(∑CEM43) (Figure 3C). For SH-SY5Y cells, the maximum co-expression of the three ICD correlates was attained at thermal doses between 5.4–10 log(∑CEM43). 

Similarly, PBNP-PTT generated a thermal dose-dependent decrease in intracellular ATP of LAN-1 cells, suggesting its release (Figure 3D), with <10% intracellular ATP remaining after treatment with a thermal dose of 11.3 log(∑CEM43). Concurrently, PBNP-PTT generated an increase of released HMGB1 at thermal doses ≥1.8 log(∑CEM43), with the maximal release of HMGB1 occurring at a thermal dose of 5.1 log(∑CEM43) (Figure 3E). PBNP-PTT also caused an increase in LAN-1 cell surface calreticulin expression in a thermal dose-dependent manner, with the maximal expression of calreticulin observed upon treatment with a thermal dose of 11.3 log(∑CEM43) (Figure 3F). For LAN-1, the maximum co-expression of the three ICD correlates was attained at a thermal dose of 10.0 log(∑CEM43). Please see the flow cytometric gating strategy in Figure S3. Together, these data show that thermal doses of ≥10.0 log(∑CEM43) represent the optimal thermal dose window for maximal ICD markers in both SH-SY5Y and LAN-1 neuroblastoma cells.

### 3.3. PBNP-PTT Changes the Expression of Immunogenic Markers on Human Neuroblastoma Cells In Vitro

**Figure 4:**
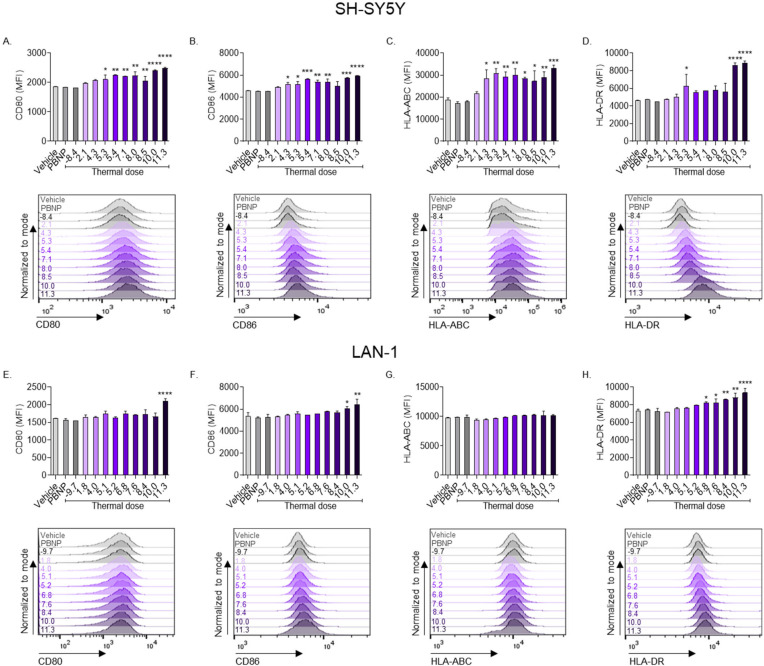
The effect of PBNP-PTT-administered thermal dose on co-stimulatory and HLA molecules is more pronounced in SH-SY5Y cells compared with LAN-1 cells. (**A**–**D**) SH-SY5Y and (**E**–**H**) LAN-1 cells were subjected to various thermal doses via PBNP-PTT. After treatment, cells were rested for 24 h and evaluated by flow cytometry analysis for (**A**,**E**) CD80, (**B**,**F**) CD86, (**C**,**G**) HLA-ABC, and (**D**,**H**) HLA-DR. MFI is represented as bar graphs representing averages, and corresponding representative histograms. Inset values in the histograms denote the thermal dose. Each parameter was compared to the vehicle control by one-way ANOVA, where * *p* < 0.03, ** *p* < 0.002, *** *p* < 0.0002, **** *p* < 0.0001; *n* = 2/group.

In addition to ICD, we investigated the effect of the thermal doses generated by PBNP-PTT to upregulate molecules involved in immune cell co-stimulation and antigen presentation on neuroblastoma cells in vitro. PBNP-PTT generated significant thermal dose-dependent increases in the expression of several measured markers. A thermal dose of 11.3 log(∑CEM43) generated the maximum expression of CD80 (maximum MFI (mMFI): 2490; Figure 4A), CD86 (mMFI: 5931; Figure 4B), HLA-ABC (mMFI: 33153; Figure 4C), and HLA-DR (mMFI: 8859; Figure 4D) on SH-SY5Y cells, suggesting an increase in the immunogenic potential of the cells in response to treatment. Similarly, a thermal dose of 11.3 log(∑CEM43) induced the greatest expression of CD80 (mMFI: 2096; Figure 4E), CD86 (mMFI: 6440; Figure 4F), and HLA-DR (mMFI: 9378; Figure 4H) on LAN-1 cells, although these changes were reduced compared with that measured in SH-SY5Y cells. HLA-ABC expression on LAN-1 cells was unchanged after treatment with all PBNP-PTT thermal doses tested (Figure 4G).

**Figure 5:**
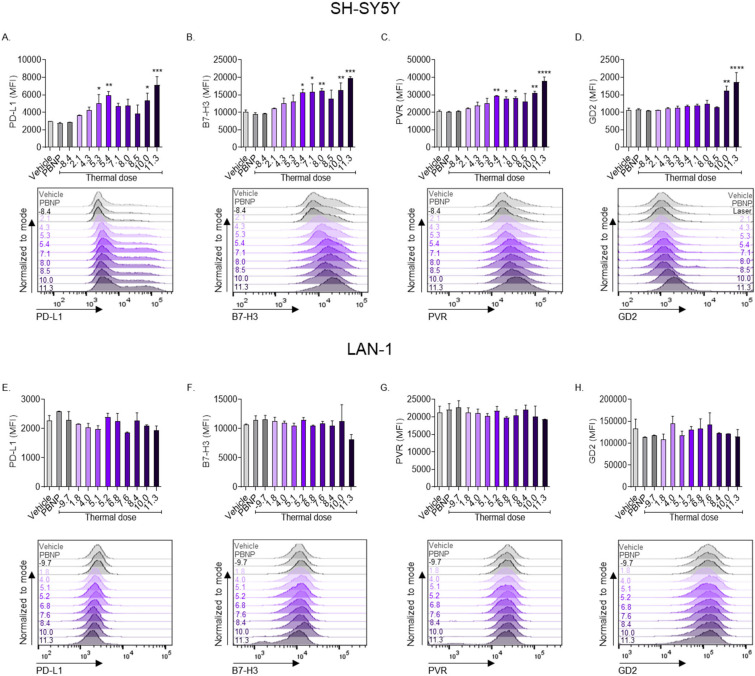
The effect of PBNP-PTT-administered thermal dose on immune checkpoint molecules, an NK cell-activating ligand, and a neuroblastoma-associated antigen is more pronounced in SH-SY5Y cells compared with LAN-1 cells. (**A**–**D**) SH-SY5Y and (**E**–**H**) LAN-1 cells were treated in vitro with varied thermal doses via PBNP-PTT. 24 h post-PBNP-PTT, cells were analyzed for expression of (**A**,**E**) PD-L1, (**B**,**F**) B7-H3, (**C**,**G**) PVR, and (**D**,**H**) GD2 using flow cytometry analysis. Inset values in the histograms denote the thermal dose. Each parameter was compared to the vehicle control by one-way ANOVA, where * *p* < 0.03, ** *p* < 0.002, *** *p* < 0.0002, **** *p* < 0.0001; *n* = 2/group.

To further investigate the immunogenic effects of thermal doses generated by PBNP-PTT on neuroblastoma cells in vitro, we measured the expression of immune checkpoint molecules and an NK cell activating ligand in response to treatment. SH-SY5Y cells increased expression of measured molecular markers in a thermal dose-dependent manner. A thermal dose of 11.3 log(∑CEM43) generated the maximum expression of PD-L1 (mMFI: 7116; Figure 5A), B7-H3 (mMFI: 19799; Figure 5B), and PVR (mMFI: 37880; Figure 5C). None of the tested thermal doses generated significant changes in expression of PD-L1, B7-H3, or PVR in LAN-1 cells (Figure 5E–G).

To measure the effect of thermal dose on antigenicity of neuroblastoma cells in vitro, we measured the expression of GD2 on the surface of the cells after treatment with PBNP-PTT at varied thermal doses. GD2 was significantly increased on SH-SY5Y cells at thermal doses ≥10 log(∑CEM43), with the maximal expression occurring at a thermal dose of 11.3 log(∑CEM43) (mMFI: 1858; Figure 5D), suggesting an increase in the antigenicity of the surviving SH-SY5Y cells in response to treatment. GD2 expression on LAN-1 cells was unchanged compared to vehicle treatment at all applied thermal doses (Figure 5H). Overall, SH-SY5Y cells upregulated co-stimulatory molecules, immune checkpoint molecules, major histocompatibility complexes, an NK cell-activating ligand, and a neuroblastoma-associated antigen in a thermal dose-dependent manner. LAN-1 cells upregulated co-stimulatory molecules (CD80 and CD86) and one major histocompatibility complex (HLA-DR) in a thermal dose-dependent manner, but other immunogenic markers were unchanged in response to PBNP-PTT.

### 3.4. PBNP-PTT Impacts the Immunogenicity of SH-SY5Y Cells More than LAN-1 Cells

**Figure 6:**
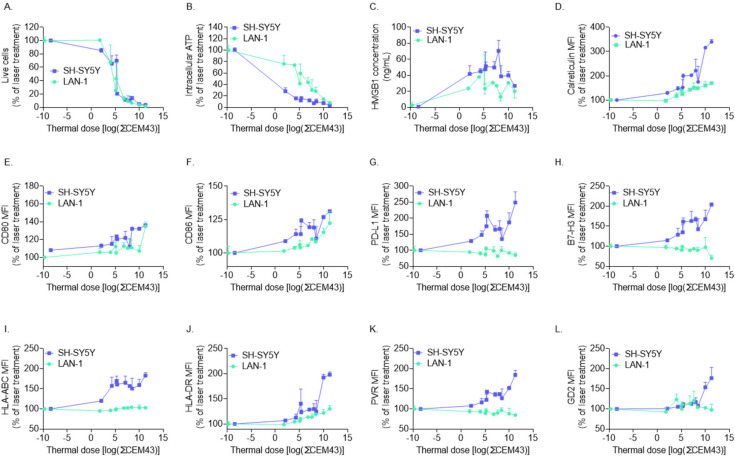
PBNP-PTT triggers greater immunophenotypic changes in MYCN-non-amplified SH-SY5Y cells than MYCN-amplified LAN-1 neuroblastoma cell line in vitro. SH-SY5Y (blue) and LAN-1 (green) cells were treated with varied thermal doses via PBNP-PTT and analyzed for (**A**) % live cells (**B**) intracellular ATP, (**C**) secreted HMGB1, and cell surface expression levels of (**D**) calreticulin, (**E**) CD80, (**F**) CD86, (**G**) PD-L1, (**H**) B7-H3, (**I**) HLA-ABC, (**J**) HLA-DR, (**K**) PVR, and (**L**) GD2. Data represent mean ± SD (*n* = 2 independent samples).

To evaluate the immunogenic changes occurring upon treatment with PBNP-PTT at varied thermal doses across neuroblastoma cell lines with different MYCN amplification statuses, we compared the effects of thermal dose on SH-SY5Y cells versus LAN-1 cells. Although the remaining tumor cells (%live cells) were similar for both SH-SY5Y and LAN-1 cells as a function of thermal dose (Figure 6A), ICD marker expression was higher in SH-SY5Y cells than LAN-1 cells as measured by decreased intracellular ATP (Figure 6B), increased extracellular HMGB1 (Figure 6C), and higher calreticulin expression (Figure 6D) at each thermal dose. Furthermore, co-stimulatory molecule expression (CD80; Figure 6E and CD86; Figure 6F), immune checkpoint molecule expression (PD-L1; Figure 6g and B7-H3; Figure 6H), both classes of major histocompatibility complexes (HLA-ABC; Figure 6I and HLA-DR; Figure 6J), NK cell-activating ligand (PVR; Figure 6K), and neuroblastoma-associated antigen expression (GD2; Figure 6L) were consistently increased to a greater extent in SH-SY5Y cells than LAN-1 cells, as illustrated by the greater slopes of the SH-SY5Y thermal dose versus immunogenic marker graphs in comparison with LAN-1. These data suggest that SH-SY5Y cells are more immunogenically responsive to changes in PBNP-PTT thermal dose than LAN-1 cells.

### 3.5. Increased Immunogenicitiy Generated by Equivalent Thermal Doses in SH-SY5Y Cells Relative to LAN-1 Cells Elicits Higher T Cell Cytotoxicity 

**Figure 7:**
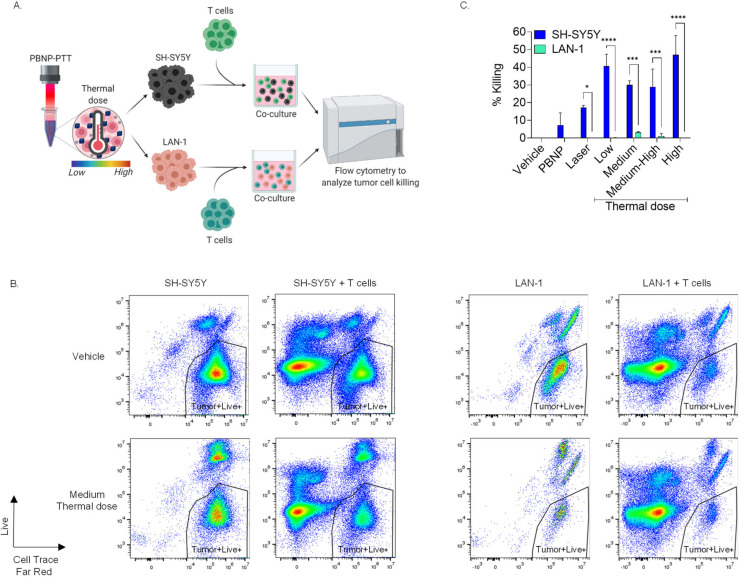
Equivalent thermal doses of PBNP-PTT induces greater T cell cytotoxicity against SH-SY5Y tumor cells than LAN-1 cells. Counterclockwise (**A**) Schematic representing the workflow of the co-culture study. SH-SY5Y and LAN-1 cells were treated with either control treatments or PBNP-PTT treatments at varied thermal doses (low to high). Tumor cells were then co-incubated with their respective tumor-reactive T cells at a 1:1 effector:target cell (E:T) ratio for 4 h and were analyzed for T cell cytotoxicity toward the treated tumor cells using flow cytometry. (**B**) Scatter plots showing live target cells for a representative thermal dose treatment group as compared with the control. (**C**) Cytotoxicity of T cells toward tumor cells when exposed to various (low to high) thermal doses (SH-SY5Y- low: 5.1, medium: 7.2, medium-high: 9.9, high: 11.2; LAN-1- low: 4.2, medium: 6.9, medium-high: 10.1, high: 11.5). Error bars depict standard deviation between two donors. Studies with each donor were performed in duplicate. A two-way ANOVA was used to compare the statistical significance between the two tumor lines (* *p* < 0.03, *** *p* < 0.0002, **** *p* < 0.0001).

To confirm whether SH-SY5Y cells are more immunogenically responsive than LAN-1 cells to changes in PBNP-PTT thermal dose, we conducted functional assays with T cells. In these studies, T cells, which were previously primed with DCs pulsed with either SH-SY5Y or LAN-1 lysates as previously published [43], were respectively co-cultured with SH-SY5Y or LAN-1 cells treated with varying PBNP-PTT thermal doses (ranging from low to high; Figure 7C). The cytotoxicity of T cells toward PBNP-PTT-treated SH-SY5Y cells was significantly higher than that toward LAN-1 at all thermal doses tested (Figure 7B,C and Figure S4). At a low PBNP-PTT thermal dose (4.2–5.1), the %killing by T cells was significantly higher for SH-SY5Y cells (40%) compared with no killing of LAN-1 cells. Similarly, at the medium, medium-high, and high thermal doses, the %killing of tumor cells by T cells was 30% (medium), 29% (medium-high), 47% (high), and 3% (medium), and 1% (medium-high) and 0% (high) for SH-SY5Y and LAN-1 cells, respectively. These findings of significantly increased cytotoxicity of T cells toward PBNP-PTT-treated SH-SY5Y cells relative to LAN-1 cells over equivalent ranges of thermal doses tested support earlier observations that PBNP-PTT thermal doses generate increased immunogenicity correlates in SH-SY5Y tumor cells compared with high-risk LAN-1 cells. 

## 4. Discussion

In this study, we presented a methodology to assess the immunogenicity elicited in tumor cells treated with varying thermal doses of PBNP-PTT by measuring specific phenotypic markers. The induction of immunogenicity and ICD, in addition to cytotoxicity, is a desirable function of a cancer therapeutic, as it offers the potential for durable and persistent anti-tumor immune effects and favorable clinical responses [22,44]. Prior literature established the biochemical correlates required to define a cell as undergoing ICD [23], that is, the release of ATP and HMGB1 from the cell, and the exposure of calreticulin on the cell surface. Here, we observed that thermal doses >5 log(∑CEM43) induced maximal levels of ICD in SH-SY5Y cells, and thermal doses of ≥10 log(∑CEM43) in LAN-1 cells, as measured by decreased intracellular ATP, increased extracellular HMGB1, and increased cell surface calreticulin expression (Figure 3). Thus, a higher thermal dose is required to generate ICD in *MYCN*-amplified LAN-1 cells than in *MYCN-*non-amplified SH-SY5Y cells.

Furthermore, PBNP-PTT at thermal doses ≥5.3 log(∑CEM43) generated significantly increased expression of both CD80 and CD86 co-stimulatory molecules on SH-SY5Y cells (Figure 4A,B). CD80 and CD86 were also increased on LAN-1 cells upon treatment with PBNP-PTT, but this only occurred at thermal doses ≥11.3 log(∑CEM43), again requiring increased heating to induce these immunogenic changes (Figure 4E,F). CD80 and CD86 are critical to the function of T cells [45,46] and are not typically present on the surface of neuroblastoma cells [47]. Thus, by increasing their expression by applying particular thermal doses to the cells via PBNP-PTT, T cell engagement and activation may be increased.

Both HLA-ABC and HLA-DR were significantly increased on SH-SY5Y cells upon PBNP-PTT administration (Figure 4C,D); HLA-DR was significantly increased on LAN-1 cells at several thermal doses tested, but HLA-ABC expression remained unchanged on LAN-1 cells at all thermal doses tested, in comparison with vehicle treatment (Figure 4G,H). These findings suggest the potential engagement of both CD4+ and CD8+ T cells with HLA-DR and HLA-ABC on SH-SY5Y cells, respectively, but not LAN-1 cells. Downregulation of HLA-ABC and HLA-DR represents a key strategy for cancer cell immune evasion. This downregulation or absence has been observed in many cancers, including neuroblastoma [48,49]. Applying specific thermal doses via PBNP-PTT offers a potential strategy for recovering immune surveillance of both *MYCN*-non-amplified and, to a lower extent, *MYCN-*amplified neuroblastoma by upregulating HLA-ABC and/or HLA-DR.

Additionally, PVR has been shown to critically determine NK cell-mediated cytotoxicity of neuroblastoma cells [50,51]. As such, upregulation of PVR in *MYCN-*non-amplified SH-SY5Y neuroblastoma cells by PBNP-PTT-administered thermal doses (Figure 5C) may enable increased NK cell-mediated tumor clearance.

In addition, the success of combining PTT with complementary immunotherapies may depend on the application of PTT at the thermal dose optimal for generating immunogenicity. The immune checkpoint molecule B7-H3 is widely expressed on neuroblastoma cells [52,53] and is the target of several strategies including B7-H3-specific chimeric antigen receptor (CAR) T cell therapy [54,55,56]. Thus, although only SH-SY5Y cells increased B7-H3 expression in response to PBNP-PTT (Figure 5F), the basal B7-H3 expression on LAN-1 cells may be adequate for targeting with B7-H3-specific treatment strategies in combination with PTT.

Anti-GD2 immunotherapy has emerged as a promising therapy for high-risk neuroblastoma [57,58]. Although LAN-1 cell surface GD2 expression was unchanged from baseline in response to PBNP-PTT (Figure 5H), GD2 was significantly increased on the surface of SH-SY5Y cells after treatment with several PBNP-PTT-administered thermal doses (Figure 5D). This effect suggests the potential combination therapy of anti-GD2 immunotherapy with PBNP-PTT for *MYCN*-non-amplified neuroblastoma. Another promising combination therapy is PBNP-PTT combined with anti-PD-L1 immunotherapy. Anti-PD-L1 monoclonal antibody therapy is clinically approved for several types of cancer [59,60], and may combine effectively with the PD-L1 upregulation on *MYCN-*non-amplified neuroblastoma cells resulting from PBNP-PTT treatment (Figure 5A).

*MYCN* amplification and MYC expression have been shown to correlate with a neuroblastoma tumor microenvironment low in infiltrating immune cells and low cellular immunity, suggesting its role in suppressing immunogenicity [61,62,63]. Our findings in vitro appeared to confirm these observations. In particular, T cells co-cultured with PBNP-PTT-treated SH-SY5Y (*MYCN*-non-amplified) and LAN-1 (*MYCN*-amplified) exhibited significantly higher cytotoxicity toward SH-SY5Y cells compared with LAN-1 cells, where minimal to negligible tumor cell killing by T cells was observed (Figure 7). Interestingly, targeting MYC has been shown to induce tumor cell immunogenicity [62]. As such, combining PBNP-PTT with MYC targeting via drugs such as I-BET726 or JQ1 in *MYCN-*amplified neuroblastoma cells may generate immunogenicity more similar to that seen in SH-SY5Y cells, and may provide effective treatment for high-risk neuroblastoma patients with *MYCN* amplification.

## 5. Conclusions

This study illuminated a consistent method for generating a range of thermal doses upon administration of PBNP-PTT to neuroblastoma cells, by varying the PBNP concentration and incident laser power. The data suggest a differential immunogenic effect of PBNP-PTT on neuroblastoma cells in vitro, perhaps correlative with *MYCN* amplification and/or risk stratification. *MYCN*-non-amplified SH-SY5Y cells underwent ICD and upregulated markers associated with immunogenicity in response to several thermal doses via PBNP-PTT. *MYCN-*amplified LAN-1 cells, although they underwent ICD, only upregulated few markers associated with immune cell engagement, compared with SH-SY5Y cells, and these responses occurred at higher thermal doses than in SH-SY5Y cells, and to a lower extent. Co-culture studies with T cells, where significantly increased cytotoxicity was observed toward SH-SY5Y cells, confirmed the increased immunogenicity of SH-SY5Y cells relative LAN-1 cells at equivalent thermal doses. Future studies will investigate the role of p53 mutation on the differential immunogenicity observed between the two cell lines. These data provide a foundation to investigate the role of *MYCN* in the immunogenic potential elicited by PBNP-PTT on neuroblastoma cells, as the mechanism driving these effects was not investigated here. This report also underscores the importance of tumor cell type and tumor cell-specific thermal dose in determining the efficacy of PBNP-PTT, both as a monotherapy and in combination with immunotherapies, for not only neuroblastoma but also other envisioned oncology indications.

## Data Availability

The data presented in this study are available within the article and Supplementary Materials.

## Supplementary Materials

The following are available online at https://www.mdpi.com/article/10.3390/cancers14061447/s1. Figure S1: Physical properties of PBNPs, Figure S2: PBNP-PTT decreases SH-SY5Y and LAN-1 cell viability in a thermal dose-dependent manner, Figure S3: Representative flow cytometry gating strategy used in the study, and Figure S4: T cell-mediated lysis is more effective against PBNP-PTT-treated SH-SY5Y tumor cells relative to LAN-1 tumor cells at equivalent thermal doses.
