# Supplementary material for: The Thermal Dose of Photothermal Therapy Generates Differential Immunogenicity in Human Neuroblastoma Cells
Source: Cancers (Basel). 2022 Mar 11;14(6):1447. doi: 10.3390/cancers14061447 (PMC8945975; doi:10.3390/cancers14061447)
Supplement: Supplementary file 1 [file cancers-14-01447-s001.zip › cancers-1522161-supplementary.pdf]

cancers

MDPI

Supplementary materials

The Thermal Dose of Photothermal Therapy Generates Differential Immunogenicity in Human Neuroblastoma Cells

Palak Sekhri [1], Debbie K. Ledezma [1,2], Anshi Shukla [1], Elizabeth E. Sweeney [3,*] and Rohan Fernandes [1,2,*]

[1] George Washington Cancer Center, Department of Medicine, School of Medicine and Health Sciences, George Washington University, Washington, DC, USA; palaksekhri@gwu.edu (P.S.); dkledezma@gwmail.gwu.edu (D.K.L.); anshi.shukla1610@gmail.com (A.S.)
[2] The Institute for Biomedical Sciences, School of Medicine and Health Sciences, George Washington University, Washington, DC, USA
[3] George Washington Cancer Center, Department of Biochemistry & Molecular Medicine, School of Medicine and Health Sciences, George Washington University, Washington, DC, USA
* Correspondence: lizie@gwu.edu (E.E.S.); rfernandes@gwu.edu (R.F.)

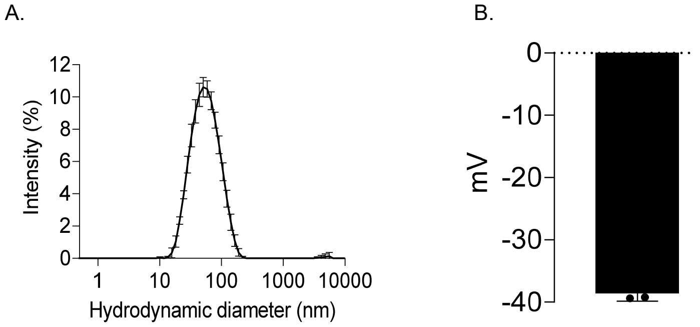

Figure S1. Physical properties of PBNPs. PBNPs were synthesized and assessed for their physical properties using dynamic light scattering. (A) The size distribution of the PBNPs was measured with an average hydrodynamic diameter of 55 nm and a polydispersity index (PDI) of 0.2. (B) PBNPs had a mean surface charge of -39 ± 1.3 mV as measured by their zeta potential.

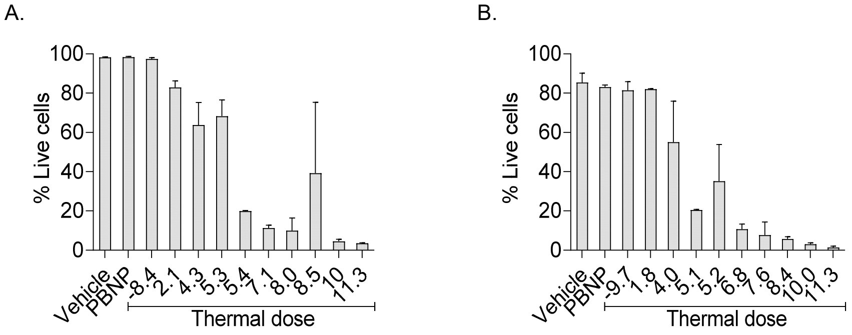

Figure S2. PBNP-PTT decreases SH-SY5Y and LAN-1 cell viability in a thermal dose-dependent manner. (A) SH-Scheme 5. Y and (B) LAN-1 cells were subject to PBNP-PTT at different thermal doses. After 24 h, cells were analyzed for viability using flow cytometry. Graphs represents the percentage of live cells for each condition using a live-dead stain.

 https://doi.org/10.3390/cancers14061447 https://www.mdpi.com/journal/cancers

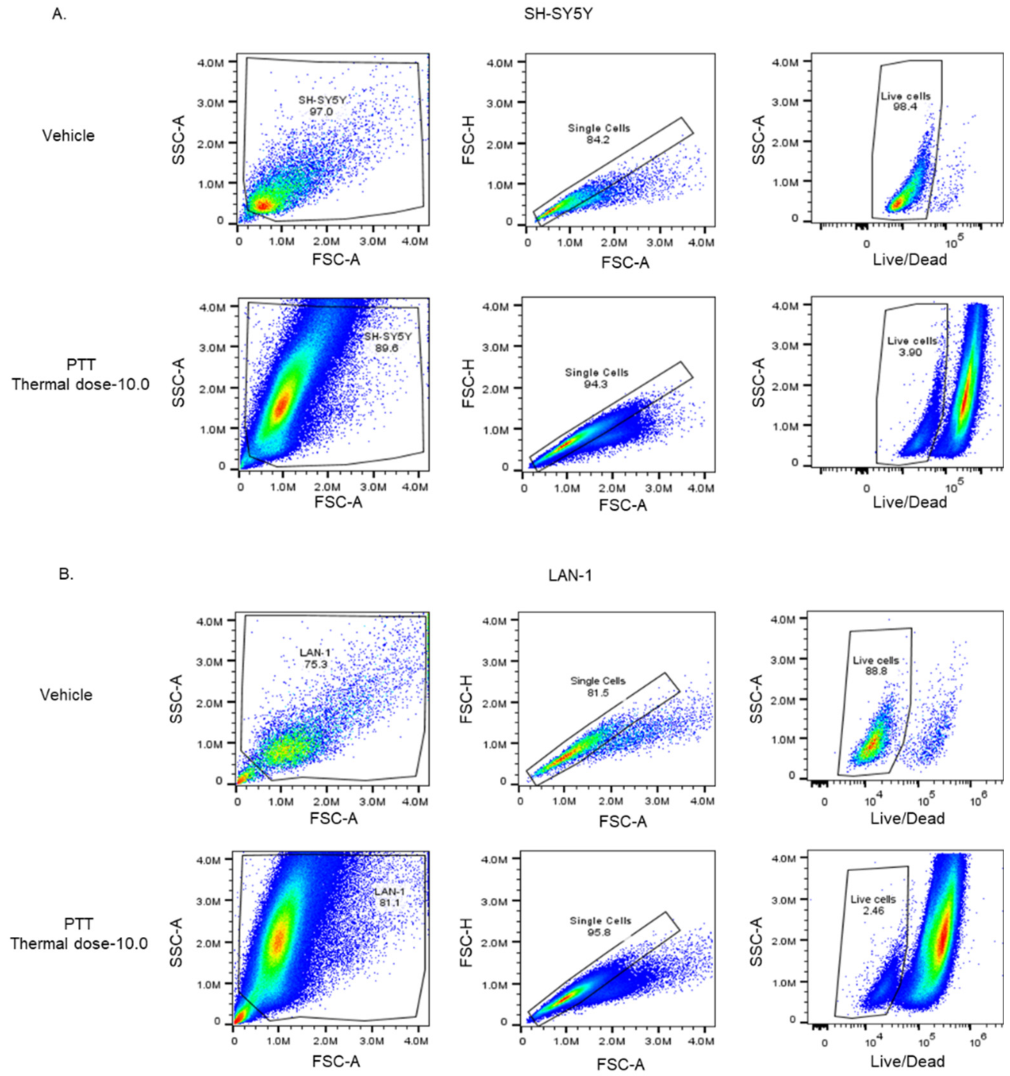

Figure S3. Representative flow cytometry gating strategy used in the study. Representative scatter plots illustrates the gating strategy for flow cytometric analysis for (A) SH-SY5Y and (B) LAN-1 for vehicle (control) and PBNP-PTT at a thermal dose of 10.0 log(CEM43).

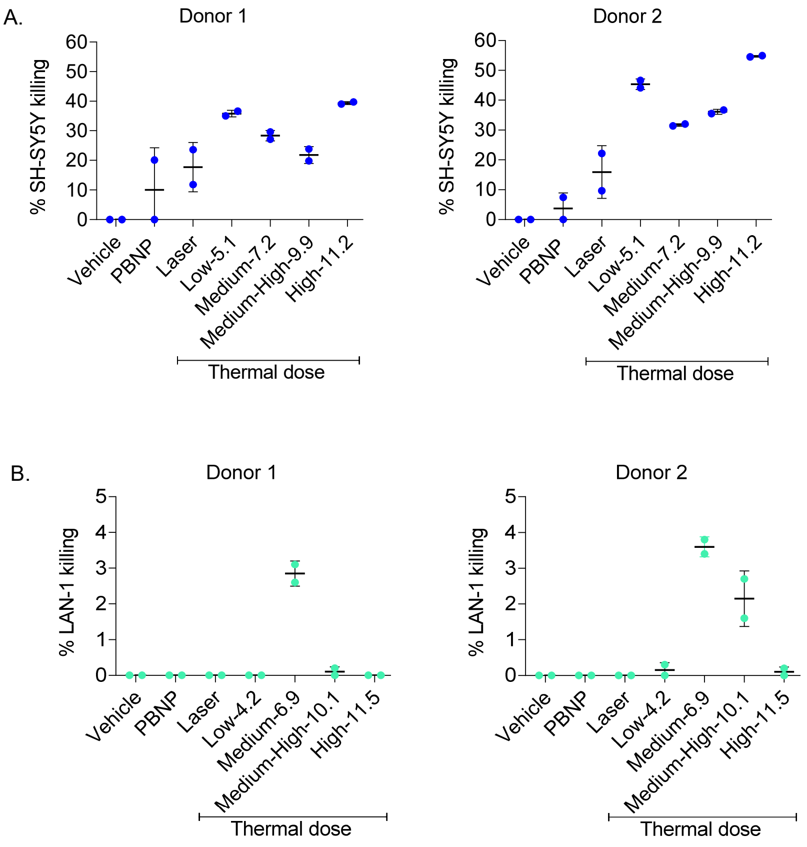

Figure S4. T cell-mediated lysis is more effective against PBNP-PTT-treated SH-SY5Y tumor cells relative to LAN-1 Table 4. h, the cells were stained and flow cytometry was performed to analyze T cell cytotoxicity against tumor cells. % Killing of target tumor cells by the tumor lysate-reactive T cells for (A) SH-SY5Y (top) and (B) LAN-1 (bottom) in two donors. PBNP-PTT was administered using either 0.1 mg/mL or 0.15 mg/mL PBNP. The error bars denote standard deviation between replicates for each donor.
